# Association of rs2435357 and rs2506030 polymorphisms in *RET* with susceptibility to hirschsprung disease: A systematic review and meta-analysis

**DOI:** 10.3389/fped.2022.1030933

**Published:** 2022-10-17

**Authors:** Jianhua Mu, Yuxi Zhang, Guoying Liao, Xinxin Li, Yinyan Luo, Zhaorong Huang, Caiyun Luo, Kai Wu

**Affiliations:** ^1^ The Second School of Clinical Medicine, Southern Medical University, Guangzhou, Guangdong, China; ^2^ Department of Pediatric Surgery, Zhujiang Hospital, Southern Medical University, Guangzhou, Guangdong, China

**Keywords:** hirschsprung disease, meta-analysis, susceptibility, single nucleotide polymorphism, RET

## Abstract

**Background:**

There are numerous published studies on the association between *RET* polymorphisms and susceptibility to Hirschsprung disease (HSCR). However, some of the results are inconsistent and the studies were conducted with small sample sizes. Therefore, we performed a meta-analysis to clarify the relationship.

**Methods:**

Relevant data were retrieved from PubMed, Web of Science, Cochrane Library, EMBASE, CNKI, and Google Scholar according to PRISMA guidelines. Odds ratios (OR) were calculated to assess susceptibility to HSCR. Meanwhile, heterogeneity and publication bias were also calculated by R software package (version 4.2.1). The protocol was published in PROSPERO (CRD42022348940).

**Results:**

A total of 12 studies were included in the meta-analysis and comprised 12 studies on the *RET* polymorphism rs2435357 (1,939 subjects and 3,613 controls) and 7 studies on the *RET* polymorphism rs2506030 (1,849 patients with HSCR and 3,054 controls). The analysis revealed that rs2435357 [A vs. G: odds ratio (OR) = 3.842, 95% confidence interval (CI) 2.829–5.220; AA vs. GG: OR = 2.597, 95% CI 1.499–4.501; AA + AG vs. GG: OR = 6.789, 95% CI 3.0711–14.9973; AA vs. AG + GG: OR = 8.156, 95%CI 5.429–12.253] and rs2506030 (A vs. G: OR = 0.519, 95% CI 0.469–0.573; AA vs. GG: OR = 0.543, 95% CI 0.474–0.623; AA + AG vs. GG: OR = 0.410, 95% CI 0.360–0.468; AA vs. AG + GG: OR = 0.361, 95%CI 0.292–0.447) were significantly associated with susceptibility to HSCR.

**Conclusions:**

The polymorphisms rs2435357 and rs2506030 in the *RET* may be related to susceptibility to HSCR, of which rs2435357 (T > C) is the causal locus and rs2506030 (A > G) is the protective locus.

**Systematic Review Registration:**

https://www.crd.york.ac.uk/prospero/, identifier:CRD42022348940.

## Introduction

Hirschsprung disease (HSCR) is a very common developmental deformity of the digestive tract with a global incidence of approximately 1 case per 5,000 live births (1). The highest incidence is in Asian populations (approximately 2.8 cases per 10,000 live births) (2). HSCR is more common in males compared with females, with a ratio of approximately 4:1 (3). The main etiology of HSCR is the dysfunction of ectodermal neural crest cells in the migration process, resulting in a lack of ganglion cells in the myenteric nerve plexus of the distal intestinal wall (4), which causes abnormal intestinal function and morphology (5). Based on the extent of aganglionosis, HSCR is classified into the total colon aganglionic type (TCA: 5%), the long segment type (L-HSCR: 15%), and the short segment type (S-HSCR: 80%) (6). Delayed meconium excretion occurs in the majority of neonatal HSCR cases, and patients present with malnutrition and developmental delay as they get older. Delayed treatment can affect a child's development and can even be life threatening.

There is increasing evidence that genetic factors play an important role in the pathogenesis of HSCR (2, 7, 8). With the extensive application of high-throughput sequencing and genotyping technology, genome-wide association studies (GWAS) are facilitating the discovery of susceptibility genes for a variety of complex diseases, including HSCR (9).

The gene *RET* is the main susceptible gene in HSCR (10). *RET* is located on the long arm of chromosome 10, consists of 21 exons, and encodes a tyrosine kinase receptor superfamily protein containing 1,114 amino acids. *RET* is activated by ligands of the glial cell line derived neurotrophic factor (GDNF) family and participates in the proliferation, migration and differentiation of enteric neural crest cells (ENCCs) (11, 12). Abnormal expression of *RET* gene leads to abnormal colonization of ENCCs in the intestinal tract, which promotes apoptosis of ENCCs and causes the occurrence of HSCR (13).

A large number of case-control studies have investigated the association between single nucleotide polymorphisms (SNPs) and HSCR susceptibility (14–16). However, some SNPs still lack a corresponding meta-analysis. *RET* gene-related mutations are associated with the pathogenesis of HSCR (17). Rs2435357 and rs2506030 have been found to be closely related to HSCR (18). Rs2435357 (T > C) is the causal locus, while rs2506030 (A > G) is the protective locus. Numerous studies on the susceptibility analysis of HSCR with rs2435357 and rs2506030 have been published. The existing meta-analysis data of rs2435357 need to be updated, and there is no meta-analysis of rs2506030 and the results of its association with HSCR susceptibility are controversial. Therefore, revealing the degree of association between rs2435357 and rs2506030 with HSCR susceptibility *via* meta-analysis will provide the possibility to further study the pathogenesis of HSCR.

## Methods

This systematic review and meta-analysis study was performed in accordance with the guidelines of the Preferred Reporting Items for Systematic Reviews and Meta-Analyses (PRISMA). The protocol was published in PROSPERO (https://www.crd.york.ac.uk/prospero/, CRD42022348940).

### Literature search strategy

The retrieval work was independently completed by two researchers through PubMed, Web of Science, Cochrane Library, EMBASE, Chinese National Knowledge Infrastructure (CNKI), and Google Scholar. Search terms were [(HSCR) or (Hirschsprung) or (HD) or (congenital megacolon)] and [(ret) or (rs2435357) or (rs2506030)], and relevant data were manually searched according to specified standards. All studies considered potentially relevant were read in their entirety and subsequently selected for inclusion in the current study. Any differences arising therein were resolved after discussion with a third researcher. Finally, the selected studies were summarized.

### Inclusion and exclusion criteria

Criteria were developed for the screening process to ensure the stability of the included data. Inclusion criteria: (1) analyzing the relationship between rs2435357 or rs2506030 and HSCR susceptibility, (2) cohort or case-control study, and (3) data for each genotype could be collected. Exclusion criteria: (1) the genotype frequency of the control group in the study did not meet the Hardy–Weinberg equilibrium (HWE, *P *< 0.01), 2) review articles, (3) animal experiments, (4) studies with duplicate data, and (5) severe data loss.

### Data collection

Data were extracted from articles that met the inclusion criteria for analysis. This included the year of publication, region, author, HWE (*P *< 0.01), sample size, genotypic data from the cases and controls, genotypic classification, and sample source.

### Quality assessment

Two independent reviewers assessed and scored the quality of the selected studies using the Newcastle–Ottawa scale (NOS) (19). The NOS scale assessed the quality of the study through eight items in three dimensions: exposure, study population selection, and comparability. The highest possible NOS score was 9, and a score of 6 points or above was considered high-quality research.

### Statistical analysis

Meta-package and Meta-for package in R Studio were used for relevant data analysis. Odds ratios (ORs) and corresponding confidence intervals (CIs) were analyzed using the data collected to quantify the relationship between the two SNPs (rs2435357 and rs2506030) and susceptibility to HSCR. During analysis, the uppercase letters “A” and “T” were identified as the mutant alleles for rs2435357 and rs2506030, respectively, while the uppercase letters “G” and “C” were defined as wild-type alleles. The HWE for each study was assessed by Chi-square test (20). Additionally, to determine the integrity of the analysis, five genetic models were selected as the main content of data analysis; these models were allele model, dominant model, recessive model, heterozygous model, and homozygous model. Subsequently, the Q test was used to assess the heterogeneity of the data included in the study. A *P* value >0.1 indicated that the data included in the study were quite heterogeneous and suitable for the use of a random effects model; otherwise, a fixed effects model was used (21). Because heterogeneity of meta-analysis can be derived from race, genotyping method, and sample source (22–24), the data included in the study were further classified according to race and genotyping method, and then subgroup analysis was performed to identify the source of the heterogeneity. In addition, the sensitivity of the overall meta-analysis was assessed by deleting data for each single study (25).

## Results

### Study characteristics

**Figure 1:**
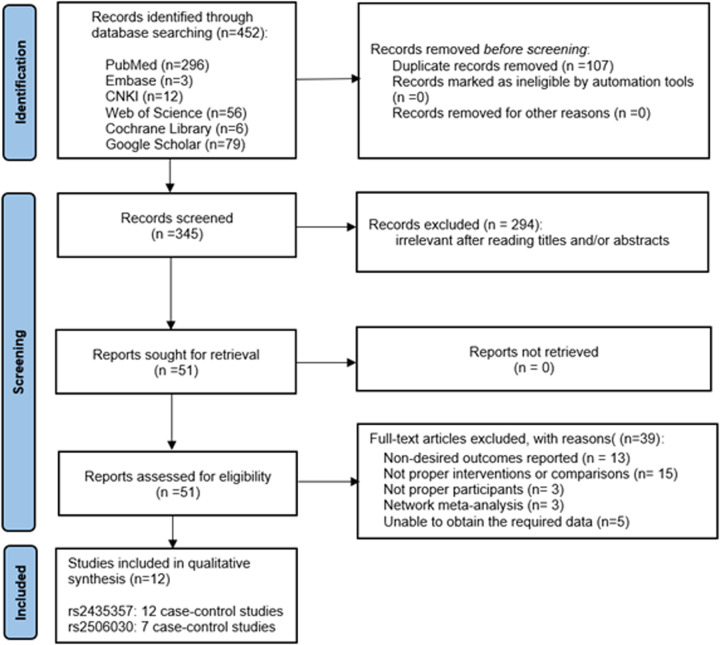
PRISMA flow diagram for literature research and data extraction.

A total of 452 studies were retrieved from PubMed, EMBASE, Web of Science and Cochrane Library, Google Scholar, and CNKI. Among them, 107 repetitive articles were excluded and 294 articles that did not meet the inclusion criteria were excluded according to the title and abstract. Subsequently, a further 39 articles were excluded after analysis of the full text of the remaining 51 articles. Ultimately, 12 articles were included in the meta-analysis. Of the 12 selected studies, 7 investigated both rs2435357 and rs2506030. A PRISMA flowchart of the literature retrieval strategy is shown in Figure 1.

There were 12 studies on rs2435357 (3, 17, 26–35) and 7 studies on rs2506030 (17, 27, 28, 31, 33–36) included in the meta-analysis. The general characteristics of the enrolled studies are presented in Table 1. Two studies were conducted in Europe (26, 30) and 10 in Asia. Only one study explored the long segment type and the short segment type of HSCR separately (28). Sample sizes ranged from 107 to 1810. Genotypic classification methods were TaqMan, Sequenom, PCR-RFLP, and PCR. Most studies were HWE balanced and three studies did not state HWE (17, 27, 28). Eight studies were hospital-based, one was population-based, and three did not state their source of controls.

**Table 1:** Main characteristics of studies included in this meta-analysis.

SNP	First Author	Ethnicity	Sample size		Cases					Controls					HWE *P* value	Source of control	Genotyping method
			Case	Control	genotypes			allele		genotypes			allele				
rs2435357					TT	CT	CC	T	C	TT	CT	CC	T	C			
	Zhang 2007	Asian	99	132	57	28	14	142	56	29	62	41	120	144	0.544	H-B	PCR
	Arnold 2008	Europe	62	30	12	27	23	51	70	2	14	14	18	42	0.542	H-B	TaqMan
	Miao 2010	Asian	315	352	228	65	22	521	109	62	169	95	293	359	0.39	H-B	PCR
	Phusantisampan 2012	Asian	68	120	47	14	7	108	28	31	64	25	126	114	0.447	H-B	PCR-RFLP
	Prato 2009	Europe	22	85	11	6	5	28	16	3	32	50	38	132	0.435	H-B	PCR
	Zhang 2015	Asian	135	118	101	31	3	233	37	13	30	16	56	62	0.88	NS	TaqMan
	Gunadi 2016	Asian	93	136	67	22	4	156	30	27	83	26	137	135	0.01	NS	PCR-RFLP
	Yang 2017	Asian	362	1448	209	126	27	544	180	329	802	317	1460	1436	0.001	*P*-B	TaqMan
	Li 2017	Asian	99	114	69	27	3	165	33	19	58	37	96	132	0.641	NS	TaqMan
	Qian Jiang 2021	Asian	120	509	88	30	2	206	34	100	252	157	452	566	0.44	H-B	Sequenom
	Yang Wang 2020	Asian	491	509	320	146	25	786	196	100	252	157	452	566	yes	H-B	Sequenom
	Kristy Iskandar 2022	Asian	73	60	62	11	0	135	11	20	34	6	74	46	yes	H-B	TaqMan
rs2506030					AA	AG	GG	A	G	AA	AG	GG	A	G			
	Gunadi 2019	Asian	60	122	3	21	36	27	93	15	50	57	80	164	yes	H-B	TaqMan
	Qian Jiang 2021	Asian	120	512	3	32	85	38	202	42	205	265	289	735	0.44	H-B	Sequenom
	DehuaYang 2017	Asian	345	1148	13	111	221	137	553	143	550	755	836	2060	yes	H-B	TaqMan
	Yang Wang 2020	Asian	502	513	15	137	346	167	829	42	205	265	289	735	yes	H-B	Sequenom
	Qian Jiang 2015	Epure	586	586	109	272	205	490	682	207	273	106	687	485	yes	NS	TaqMan
	Qi Li 2017	Asian	99	114	2	20	77	24	174	11	46	57	68	160	yes	NS	TaqMan
	Zhen Zhang 2015	Asian	137	59	3	26	108	32	242	5	22	32	32	86	yes	NS	Taqman

PCR, polymerase chain reaction restriction; PCR-RFLP, polymerase chain reaction-restriction fragment length polymorphism; H-B, hospital-based; P-B, population-based; NS, not stated; HWE, Hardy–Weinberg equilibrium.

### Research quality

All studies included in the meta-analysis were of high quality with NOS scores between 6 and 8 (Table 2). Furthermore, all the studies had a good definition and representation of cases, and all clearly described the investigation and evaluation methods. In each study, the genetic typing method of the controls and the cases was the same. However, the selection scores of the control groups were not high, and 25% of the studies did not score on this item.

**Table 2:** Newcastle–Ottawa scale (NOS) scores for the studies included in the meta-analysis.

Study	Selection				Comparability	Exposure		
	1	2	3	4	1	1	2	3
Zhang 2007	★	★	★	★	★★	★	★	
Arnold 2008	★	★	★	★	★★	★	★	
Miao 2010	★	★	★	★	★★	★	★	
Phusantisampan 2012	★	★	★	★	★★	★	★	
Prato 2009	★	★	★	★	★★	★	★	
Zhang 2015	★	★		★	★★	★	★	
Gunadi 2016	★	★		★		★	★	
Yang 2017	★	★	★	★	★★	★	★	
Li 2017	★	★		★	★★	★	★	
Qian Jiang 2021	★	★	★	★	★★	★	★	
Yang Wang 2020	★	★	★		★★	★	★	
Kristy Iskandar 2022	★	★	★	★	★★	★	★	

1. Adequate case definition; 2. Representativeness of the cases; 3. Selection of controls; 4. Definition of controls. Comparability: 1. Comparability of cases and controls on the basis of the design or analysis. Exposure: 1. Ascertainment of exposure; 2. The same method of ascertainment for cases and controls; 3: Non-response rate.

### Rs2435357

**Figure 2:**
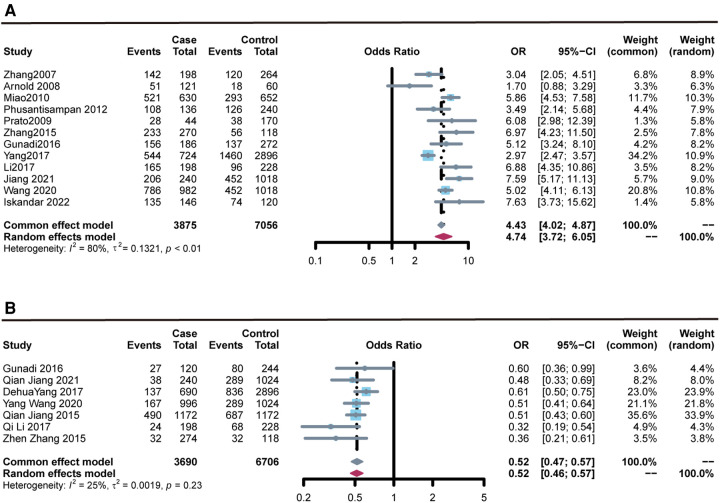
Forest plots of the association of rs2435357 and rs2506030 polymorphisms of the *RET* with susceptibility to HSCR. (**A**) rs2435357 (allele model); (**B**) rs2506030 (allele model).

The meta-analysis results of the association between the rs2435357 polymorphism of the *RET* and the susceptibility risk of HSCR are presented in Figure 2. A total of 12 studies were summarized, and 1,939 cases and 3,613 controls were included in the data analysis. The rs2435357 polymorphism was significantly associated with HSCR susceptibility risk (T vs. C: OR = 4.743, 95% CI 3.720–6.047, *P *= 3.72e-36, *I*^2^* *=* *79.8%, *P *= 0.000). In addition, the following allelic models were also significantly associated with HSCR susceptibility: homozygous (TT vs. CC: OR = 2.272, 95% CI 1.828–2.824, *P *= 2.82e-5, *I*^2^* *=* *68.0%, *P *= 0.026); dominant (TT + CT vs. CC: OR = 6.521, 95% CI 3.533–12.037, *P *= 2.21e-06, *I*^2 ^= 78.7%, *P *= 0.000); and recessive (TT + CT + CC: OR = 9.046, 95% CI 6.694–12.231, *P *= 1.52e-46, *I*^2^* *=* *76.0%, *P *= 0.000).

Stratified analysis by ethnicity revealed that the rs2435357 polymorphism was significantly associated with HSCR risk in Asia: allele mode (T vs. C: OR = 4.992, 95% CI 3.956–6.298, *P *= 7.43e-42, *I*^2 ^= 80.2%, *P *= 0.000); homozygous model (TT vs. CC: OR = 2.335, 95% CI 1.537–3.548, *P *= 7.06e-5, *I*^2 ^= 55.1%, *P *= 0.018); heterozygous model (TT vs. CT: OR = 15.655, 95% CI 9.185–26.681, *P *= 4.89e-24, *I*^2 ^= 70.2%, *P *= 0.000); dominant model (TT + CT vs. CC: OR = 7.855, 95% CI 4.028–15.320, *P *= 1.47e-09, *I*^2 ^= 79.1%, *P *= 0.000); recessive model (TT + CT + CC: OR = 8.956, 95% CI 6.596–12.161, *P *= 8.20e-45, *I*^2^* *=* *78.4%, *P *= 0.000). Subgroup analysis by genotyping method and source of controls also revealed there was association between the rs2435357 polymorphism and HSCR risk in TaqMan, PCR, and Hospital-Based (H-B) subgroups. Results for other subgroups are presented in Table 3.

**Table 3:** Analysis results of the association between rs2435357 and HSCR risk.

Subgroup	Genetic Model	Type of Model	Heterogeneity		Odds Ratio			
			I2 (%)	PH	OR	95% CI	Ztest	POR
Overall								
	T vs. C	Random	0.798	0.000	4.743	[3.720; 6.047]	12.555	3.72E–36
	TC vs. CC	Random	0.678	0.000	15.188	[9.167; 25.165]	10.560	4.55E–26
	TT vs. CC	Random	0.496	0.026	2.272	[1.828; 2.824]	4.187	2.82E–05
	TT + TC vs. CC	Random	0.787	0.000	6.521	[3.533; 12.037]	5.996	2.02E–09
	TT vs. TC + CC	Random	0.760	0.000	9.049	[6.694; 12.231]	14.325	1.52E–46
Source of controls								
HB								
	T vs. C	Random	0.732	0.000	4.617	[3.346; 6.370]	9.310	1.23E–20
	CT vs. CC	Random	0.665	0.004	13.842	[7.167; 26.732]	7.830	5.07E–15
	TT vs. CC	Random	0.587	0.018	1.946	[1.194; 3.171]	2.670	7.59E–03
	TT + CT vs. CC	Random	0.729	0.001	4.897	[2.682; 8.943]	5.170	2.33E–07
	TT vs. CT + CC	Random	0.515	0.044	8.707	[6.527; 11.615]	14.720	4.80E–49
others								
	T vs. C	Random	0.854	0.000	4.988	[3.241; 7.678]	7.300	2.80E–13
	CT vs. CC	Random	0.746	0.008	18.722	[7.453; 47.033]	6.230	4.55E–10
	TT vs. CC	Random	0.381	0.183	2.265	[1.565; 3.279]	4.330	2.02E–03
	TT + CT vs. CC	Random	0.884	0.000	11.351	[2.933; 43.939]	3.520	4.35E–04
	TT vs. CT + CC	Random	0.885	0.000	10.218	[5.140; 20.313]	6.630	3.36E–11
Genotyping methods								
TaqMan								
	T vs. C	Random	0.856	0.000	4.475	[2.563; 7.812]	5.271	1.36E–07
	CT vs. CC	Random	0.704	0.009	16.094	[5.666; 45.714]	5.216	1.83E–07
	TT vs. CC	Fixed	0.388	0.162	2.161	[1.518; 3.077]	4.270	6.64E–03
	TT + CT vs. CC	Random	0.881	0.000	9.096	[2.142; 38.630]	2.992	2.77E–03
	TT vs. CT + CC	Random	0.846	0.000	9.193	[4.724; 17.892]	6.530	6.59E–11
Others								
	T vs. C	Random	0.534	0.092	5.205	[3.965; 6.831]	11.889	1.35E–32
	CT vs. CC	Random	0.692	0.021	17.283	[6.924; 43.136]	6.106	1.02E–09
	TT vs. CC	Random	0.716	0.014	2.499	[0.972; 6.426]	1.900	5.74E–02
	TT + CT vs. CC	Random	0.705	0.017	6.639	[2.750; 16.027]	4.210	2.56E–05
	TT vs. CT + CC	Fixed	0.014	0.385	8.534	[6.768; 10.762]	18.120	2.23E–73

### Rs2506030

Data were collected from seven studies, comprising 1,849 cases with HSCR and 3,054 controls. The meta-analysis results (Figure 2) revealed that the rs2506030 polymorphism was significantly associated with susceptibility to HSCR (allele: A vs. G: *OR *= 0.519, 95% CI 0.469–0.572, *P *= 2.21E-33, *I*^2 ^= 25.5%, and *P *= 0.234). There was also sufficient evidence that the following allelic models were associated with HSCR susceptibility: homozygous (AA vs. GG: OR = 0.543, 95% CI 0.474–0.623, *P *= 7.00e-13, *I*^2 ^= 29.8%, *P *= 0.200); dominant (AA + AG vs. GG: OR = 0.410, 95% CI 0.360–0.468, *P *= 2.49e-40, *I*^2 ^= 0.00%, *P *= 0.541); and recessive (AA vs. AG + GG: OR = 0.361, 95% CI 0.292–0.447, *P *= 7.54e-17, *I*^2 ^= 0.00%, *P *= 0.803).

Subgroup analyses were performed by ethnicity, source of controls, and genotyping method. The association between *RET* gene polymorphism and HSCR susceptibility was significant in the TaqMan subgroup (A vs. G: OR = 0.525, 95% CI 0.467–0.591, *P *= 3.92e-11, *I^2 ^*= 49.0%, *P *= 0.098; AA vs. GG: OR = 0.565, 95% CI 0.477–0.670, *P *= 5.51e-6, *I^2 ^*= 49.6%, *P *= 0.094; AG vs. GG: OR = 0.274, 95% CI 0.208–0.361, *P *= 5.68e-21, *I^2 ^*= 0.00, *P = *0.851; AA + AG vs. GG: OR = 0.385, 95% CI 0.327–0.453, *P *= 2.36e-30, *I*^2^* *=* *0.00%, *P *= 0.507; AA vs. AG + GG: OR = 0.368, 95% CI 0.292–0.465, *P *= 2.16e-10, *I*^2^* *=* *0.00%, *P *= 0.593). In addition, there was an association between rs2506030 polymorphism and HSCR risk in the Hospital-Based (H-B) subgroup. Results of the subgroup analysis of sample source and genotyping method are shown in Table 4.

**Table 4:** Analysis results of the association between rs2506030 and HSCR risk.

Subgroup	Genetic Model	Type of Model	Heterogeneity		Odds Ratio			
			I2 (%)	PH	OR	95% CI	Ztest	POR
Overall								
	A vs. G	Fixed	0.255	0.234	0.519	[0.469; 0.573]	−12.039	2.21E–33
	AG vs. GG	Fixed	0.000	0.962	0.270	[0.211; 0.346]	−10.561	4.53E–26
	AA vs. GG	Fixed	0.298	0.200	0.543	[0.474; 0.623]	−7.179	7.00E–13
	AA + AG vs. GG	Fixed	0.000	0.541	0.410	[0.360; 0.468]	−13.295	2.49E–40
	AA vs. AG + GG	Fixed	0.000	0.803	0.361	[0.292; 0.447]	−8.338	7.54E–17
Source of controls								
Others								
	A vs. G	Fixed	0.475	0.149	0.475	[0.408; 0.552]	−5.390	7.06E–08
	AG vs. GG	Fixed	0.000	0.599	0.258	[0.188; 0.353]	−8.390	4.81E–17
	AA vs. GG	Fixed	0.179	0.296	0.458	[0.358; 0.585]	−4.920	8.84E–07
	AA + AG vs. GG	Fixed	0.000	0.483	0.377	[0.300; 0.475]	−8.290	1.13E–16
	AA vs. AG + GG	Fixed	0.000	0.490	0.400	[0.308; 0.518]	−6.200	5.75E–10
HB								
	A vs. G	Fixed	0.000	0.561	0.553	[0.484; 0.632]	−8.770	1.83E–18
	AG vs. GG	Fixed	0.000	0.962	0.286	[0.195; 0.420]	−6.420	1.33E–10
	AA vs. GG	Fixed	0.109	0.338	0.586	[0.497; 0.692]	−5.210	1.93E–07
	AA + AG vs. GG	Fixed	0.000	0.422	0.427	[0.364; 0.501]	−9.570	1.02E–21
	AA vs. AG + GG	Fixed	0.000	0.943	0.306	[0.210; 0.446]	−6.080	1.20E–09
Genotyping methods								
TaqMan								
	A vs. G	Fixed	0.490	0.098	0.525	[0.467; 0.591]	−6.610	3.92E–11
	AG vs. GG	Fixed	0.000	0.851	0.274	[0.208; 0.361]	−9.400	5.68E–21
	AA vs. GG	Fixed	0.496	0.094	0.565	[0.477; 0.670]	−4.540	5.51E–06
	AA + AG vs. GG	Fixed	0.000	0.507	0.385	[0.327; 0.453]	−11.450	2.36E–30
	AA vs. AG + GG	Fixed	0.000	0.593	0.368	[0.292; 0.465]	−6.350	2.16E–10
Others								
	A vs. G	Fixed	0.000	0.755	0.504	[0.418; 0.607]	−7.220	5.04E–13
	AG vs. GG	Fixed	0.000	0.764	0.262	[0.152; 0.452]	−4.820	1.41E–06
	AA vs. GG	Fixed	0.000	0.849	0.505	[0.401; 0.636]	−5.810	6.08E–09
	AA + AG vs. GG	Fixed	0.000	0.824	0.461	[0.370; 0.575]	−6.880	6.09E–12
	AA vs. AG + GG	Fixed	0.000	0.785	0.333	[0.194; 0.569]	−4.010	6.00E–05

### Sensitivity analysis

**Figure 3:**
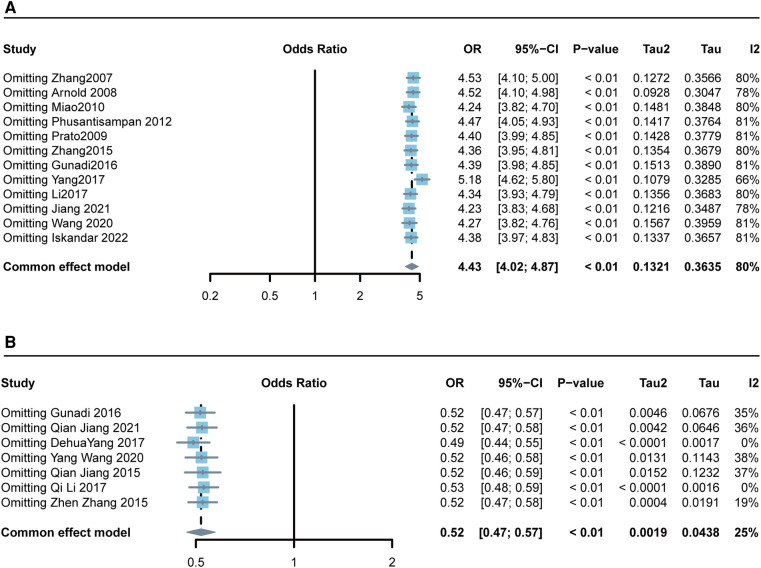
Forest plots of the sensitivity of the meta-analysis. (**A**) rs2435357 (allele model); (**B**) rs2506030 (allele model).

Sensitivity analysis calculates the overall correlation coefficient obtained by removing a group of studied data respectively, and then quantifies the difference between each correlation coefficient to obtain the stability of the whole data and the model. The robustness of the meta-analysis was assessed by comparing the results of analyses before and after the removal of one study. The processed results were not significantly different from the previous results, thus the data and model have high robustness (Figure 3).

### Publication bias

**Figure 4:**
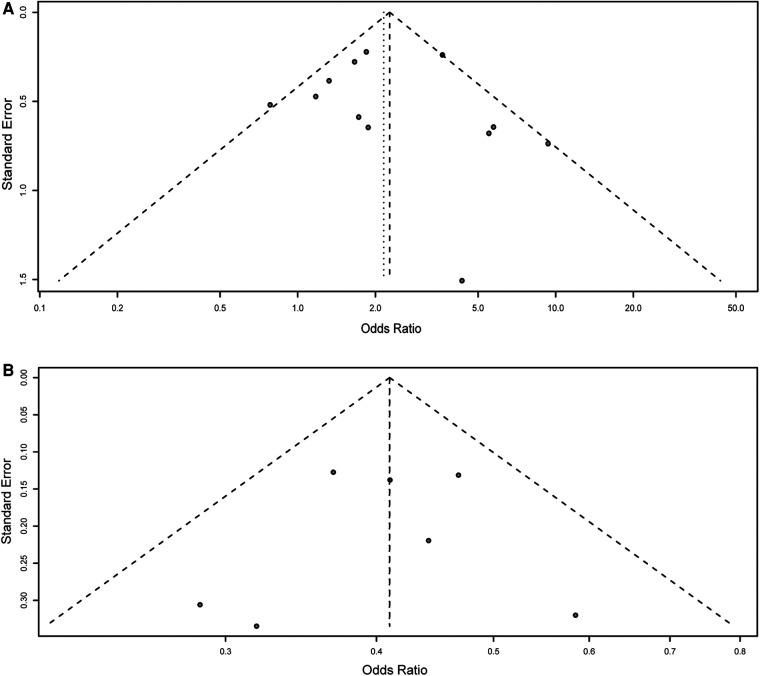
Funnel plot for the detection of the publication bias for association of rs2435357 and rs2506030 polymorphisms of the *RET* gene with HSCR risk. (**A**) rs2435357 (heterozygous model); (**B**) rs2506030 (dominant model).

Harbord's test was performed to assess the possible publication bias of included studies. The results showed that in the meta-analysis of rs2435357, five genetic models showed no publication bias: allele mode (T vs. C: *P* = 0.395); homozygous model (TT vs. CC: *P* = 0.216); heterozygous model (TT vs. CT: *P* = 0.898, Figure 4A); dominant model (TT + CT vs. CC: *P* = 0.902); recessive model (TT + CT + CC: *P* = 0.287). In addition, in the meta-analysis of rs2506030, no publication bias found: allele: A vs. G: *P* = 0.225); homozygous (AA vs. GG: *P* = 0.470); heterozygous model (AA vs. AG: *P* = 0.172); dominant model (AA + AG vs. GG: *P* = 0.732, Figure 4B); and recessive model (AA vs. AG + GG: *P* = 0.070).

## Discussion

*RET* gene is located on the long arm of chromosome 10, contains 21 exons, and encodes receptor tyrosine kinase transmembrane protein. RET protein has three different subtypes, named RET51, RET43, and RET9. Moreover, Bhattarai et al. (37) showed that interactions between RET protein and its ligands can control the survival, migration, proliferation, differentiation, and maturation of vagal and sacral neural crest cells. Nagy et al. (38) demonstrated that excessive stimulation of *RET* al. one could lead to HSCR. Mutations in the *RET* are currently detected in approximately half of familial aggregate patients with HSCR and in 7% to 35% of patients with sporadic HSCR (39–41). Using the strategy of combining gene-gene interaction analysis with case-control study, Wang et al. demonstrated for the first time that the genetic markers of *RET*, *ARHGEF3*, and *CTNNAL1* and the related genetic interaction network can change the susceptibility risk of HSCR in the Han population (33). In addition, there is sufficient evidence that *RET* plays an important role in other developmental and metabolic disorders (42).

The results of the present study are generally consistent with previous meta-analyses of *RET* polymorphisms and susceptibility to HSCR. Rs2435357 has been the focus of attention since Liang et al. (43) conducted the first meta-analysis to explore the association between *RET* polymorphisms and HSCR susceptibility in 2014. However, due to various limitations, previous meta-analyses for *RET* polymorphisms and HSCR susceptibility are problematic to differing degrees in that the sample sizes are small and the analysis indexes are shallow. To obtain accurate and effective data on the potential association, a meta-analysis with larger samples and analysis at a deeper level was needed.

Of the two SNPs included in our meta-analysis, rs2435357 is currently the most studied and the most significantly associated with HSCR susceptibility. rs2506030 is an association point for a ∼125-kb upstream of *RET*, identified by Jiang et al. (Manuscript in Review at the *American Journal of Human Genetics*) in a single GWAS using cases of HSCR of European origin (EA) from five different countries (US, Italy, France, Spain, and the Netherlands). Three SNPs—rs2435357, rs2506030, and rs2506004—were previously reported to be potentially unrelated to HSCR susceptibility (17). The results of the current study confirmed a significant association between two genetic polymorphisms—rs2435357 and rs2506030—and susceptibility to HSCR. Interestingly, not all gene models were related to HSCR risk in ethnicity subgroups and genotyping subgroups in the present study, which suggested that ethnicity and genotyping methods may influence the association of genetic polymorphisms with HSCR susceptibility. In addition, all study data were found to be from Europe and Asia during the analysis, and no studies from America and Africa meeting the inclusion criteria were available to evaluate the association between *RET* and HSCR susceptibility through meta-analysis. One of the two SNPs included in this study was a causal locus, whereas the other was a protective locus. The OR value of rs2435357 (T > C) is 3.842, suggesting that rs2435357 promotes the incidence of HSCR; while the OR value of rs2506030 (A > G) is 0.519, indicating that rs2506030 (A > G) has a positive effect on HSCR. Previous studies have found that rs2435357 and rs2506030 do not have linkage disequilibrium (44), implying that the effects of these two SNPs can be enhanced or inhabited by each other. Therefore, the purpose of further research can be achieved by detecting the mutation of rs2435357 and rs2506030.

However, the study also has defects. The first is that the sample size of the study is still small, which may affect the final results. Second, the results of this study may not be applicable to cases from other regions since the cases included in this study were only from Asia and Europe. Third, only published study data were included in the meta-analysis, which could cause publication bias. In addition, most of the articles included in the study lacked data on gender, age, and pathological type of HSCR of the subjects, therefore hierarchical analysis was performed on these covariates. Finally, HSCR is a combination of environmental and genetic aspects that cannot be comprehensively analyzed in a meta-analysis due to limitations in the study data.

## Conclusion

This study revealed the relationship between two polymorphisms of *RET*—rs2435357 and rs2506030 and HSCR susceptibility, with rs2506030 exhibiting the most significant association with HSCR susceptibility. In the future, larger samples and more comprehensive analysis will more fully determine their association. Moreover, the related findings of the association between *RET* gene polymorphisms and HSCR susceptibility provide novel suggestions for HSCR pathogenesis research.

## Data Availability

The original contributions presented in the study are included in the article/Supplementary Material, further inquiries can be directed to the corresponding author/s.
